# Flexible PAN/P25 Multi-Porous Nanotubular Electrospun Membrane Constructed by a Facile Ethylene Glycol Solvothermal Induction with Excellent Photocatalytic Degradation and Sterilization Performance

**DOI:** 10.3390/polym16243484

**Published:** 2024-12-13

**Authors:** Yiwen Miao, Chenghao Zhang, Ya Sun, Chunlei Wang, Juntao Yan, Sunhua Deng, Ruan Chi

**Affiliations:** 1 College of Chemical and Biomolecular Engineering, The Hong Kong University of Science and Technology, Hong Kong 999077, China; ymiaoaa@connect.ust.hk; 2 College of Chemistry and Environmental Engineering, Wuhan Polytechnic University, Wuhan 430023, China; sunya@whu.edu.cn (C.Z.); sunya230@whpu.edu.cn (Y.S.); 12111@whpu.edu.cn (J.Y.); 3 College of Construction Engineering, Jilin University, Changchun 130021, China; dengsh@jlu.edu.cn; 4 Hubei Three Gorges Laboratory, Yichang 443000, China; 12112@whpu.edu.cn

**Keywords:** electrospinning, multi-porous nanotubular membrane, photocatalytic degradation and sterilization, solvothermal induction

## Abstract

A series of flexible polyacrylonitrile/TiO_2_ (PAN/P25) multi-porous nanotubular membranes were successfully constructed by facile electrospinning combined with an ethylene glycol solvothermal induce strategy. The effects of P25 dosage and solvothermal time on the morphology of samples were systematically investigated, which were characterized in terms of surface morphology, microstructure, specific surface area, thermal analysis, wettability, photoelectrochemical and fluorescence spectra. Rhodamine B (RhB) and *Escherichia coli* (*E. coli*) were employed as simulated pollutants to evaluate photocatalytic degradation and antibacterial properties of the PAN/P25-3 multi-porous nanotubular membrane. The PAN/P25-3 membrane exhibited the highest photocatalytic degradation efficiency, with 96.1% degradation of RhB within 120 min under a xenon lamp light source and a photocatalytic inactivation rate of 95.8% for *E. coli* under 365 nm monochromatic light irradiation. The photocatalytic degradation mechanism of the PAN/P25-3 multi-porous nanotubular membrane for RhB was deduced from the results of 3D-EEM fluorescence and scavenger experiments of reactive species. Additionally, the cyclic photodegradation experiments demonstrated that the PAN/P25-3 membrane maintained excellent stability and photocatalytic performance after multiple degradation cycles, confirming its potential for sustainable wastewater treatment applications.

## 1. Introduction

Organic and bacterial contaminants have brought about serious water environment pollution [1]. The emission of organic dyes from the textile industry has caused health problems for living organisms [2]. Furthermore, bacterial contamination such as *Escherichia coli* (*E. coli*) in water has also resulted in potential risks to human drinking water [3]. In this case, many water treatment technologies have been developed to purify water, such as ozone disinfection, chlorination, UV irradiation, electrolysis, physical adsorption, and photocatalytic degradation [4,5,6,7]. It has been proven that solar-driven photocatalytic oxidation technology is widely explored for water purification [8]. Semiconductor-based photocatalysts have attracted extensive attention for their ability to eliminate organic pollutants, inactivate harmful bacteria, and produce clean energy [9]. This technology is considered a promising water treatment method due to the absence of sterilization by-products [10,11]. As is known, commercially available TiO_2_ (P25) nano-photocatalyst is widely used due to its high stability, low cost and eco-friendliness [12,13]. However, the P25 nano-photocatalyst is prone to agglomerate due to its high surface energy, which reduces the photocatalytic activity [14]. In addition, it is difficult to separate and recover the P25 nano-photocatalyst from the treated solution, which limits the cyclic utilization [15,16]. In order to overcome this problem, people make it easier to recycle by loading magnetic materials in photocatalysts or using electrospinning.

Due to the large aspect ratio, large specific surface area, controllable chemical composition, good morphology and high porosity [17,18,19,20], electrospinning technology is considered to be a simple, universal, economical and effective method to fabricate one-dimensional nanofiber and composite nanofiber membranes containing inorganic particles [21,22]. The electrospun polyacrylonitrile (PAN) nanofiber possesses a one-dimensional nanostructure with good chemical stability and excellent flexibility [23], which can be utilized as a substrate to immobilize inorganic nanoparticles to construct functional membrane and avoid separating the photocatalyst from water. Pan et al. have combined the different inorganic materials with electrospun PAN, making the composite materials have different properties, such as enhanced Raman scattering [24] and adsorption [25,26,27]. Chen et al. fabricated PAN/P25 nanofibers by the electrospinning method, which possessed the enhanced piezoelectric photocatalysis performance toward Rhodamine B via polar functional group engineering [28]. P25 nanoparticles can be added to the spinning solution because of their good stability. However, many P25 nanoparticles are encapsulated into the fiber during the electrospinning process, which will reduce the exposure of active sites; thus, the photocatalytic activity of P25 is weakened. Therefore, how to make P25 nanoparticles more exposed and realize facile recycling becomes a challenge. Ramasundaram et al. have fixed P25 nanoparticles on steel mesh by electric spraying combined with a high-temperature hot-pressing method (350 °C, 100 MPa) [29]. Romas et al. have immobilized P25 nanoparticles within zinc acetate/PVA nanofibers by an electrostatically modified electrospinning process, which is calcinated at 600 °C to obtain polycrystalline ZnO and ZnO/P25 [30]. These methods often require more stringent conditions or complex equipment, so it is urgent that an economical and simple method be found. 

Herein, the flexible PAN/P25 multi-porous nanotubular membranes are firstly fabricated by a facile electrospinning method combined with an ethylene glycol solvothermal induction strategy, which possesses excellent photocatalytic degradation of dyes and photocatalytic sterilization performance. We discussed the morphological changes caused by the solvothermal treatment time. Additionally, the chemical, physical and photoelectric properties of the samples were characterized. The multi-porous nanotubular structure improves the light utilization efficiency and the transmission efficiency of e^−^ and h^+^. At the same time, the flexible PAN/P25 multi-porous nanotubular membrane has stable cycle performance. This multi-porous nanotubular functional membrane will be effectively used in the field of water pollution treatment in the future.

## 2. Experimental

### 2.1. Materials

Poly (vinyl pyrrolidone) (PVP, Mw = 1,300,000) and polyacrylonitrile (PAN, Mw = 150,000) were purchased from Aladdin Regent Company, America. N,N-dimethylformamide (DMF), and Ethylene glycol (EG) were obtained from Shanghai Chemical Regent Company, China. Rhodamine B (RhB) was obtained from Tianjin Guangfu Chemical Reagents Company, China. P25 (TiO_2_) was purchased from Degussa, and *E. coli* (ATCC 8739) was provided by the research group.

### 2.2. Preparation of PAN/PVP/P25 Fiber Membrane

**Figure 1:**
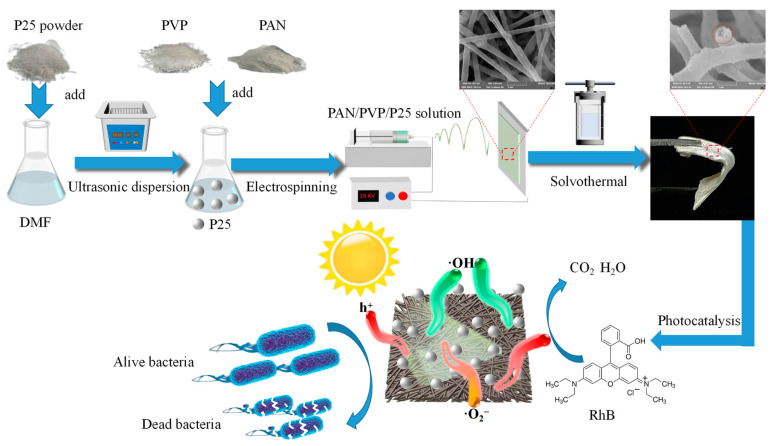
Schematic representation of the preparation and application of PAN/P25 multi-porous nanotubular membrane.

As shown in Figure 1, X g P25 (X = 0.1, 0.15, 0.2, 0.25) was ultrasonically dispersed in 6 mL DMF, then 0.6 g PAN and 0.6 g PVP were added sequentially and the uniform spinning solution was obtained after magnetic stirring for about 6 h. After that, the spinning solution was transferred to a syringe with a steel needle, which was connected to a direct current high voltage of 12 kV; the receiver was placed 15 cm away from the tip of the steel needle. The temperature and humidity were 25 °C and 45%, respectively. Finally, the as-spun fiber membrane was placed in an oven at 60 °C for 2 h to remove the residual solvent. The products were named PAN/PVP/P25-0.1, PAN/PVP/P25-0.15, PAN/PVP/P25-0.2 and PAN/PVP/P25-0.25 fiber membrane, respectively. As a control, the PAN/PVP fiber membrane was fabricated at the same conditions without introducing the P25 particles.

### 2.3. Preparation of Flexible PAN/P25 Multi-Porous Nanotubular Membrane

A flexible PAN/P25 multi-porous nanotubular membrane was achieved by the ethylene glycol (EG) solvothermal induction strategy. The as-spun PAN/PVP/P25-0.2 fiber membrane was cut into square pieces with a side length of 3 cm and transferred to the stainless steel high-pressure reactor containing EG. The reaction was performed at 180 °C for Y h (Y = 2, 3, 4). After cooling to room temperature, the obtained sample was repeatedly washed with deionized water and confronted with freeze-drying for 12 h. Finally, the PAN/P25 multi-porous nanotubular membranes were obtained, which were named PAN/P25-2, PAN/P25-3 and PAN/P25-4 multi-porous nanotubular membranes, respectively. The detailed formulation is shown in Table 1.

### 2.4. Photocatalytic Experiment

The photocatalytic activities of the obtained photocatalysts were evaluated by the degradation of RhB in an aqueous solution. The entire degradation reaction process was carried out in an opaque 100 mL jacket beaker under a light source with 2 °C condensed water throughout the jacket of the jacket beaker. Firstly, 50 mg of PAN/PVP/P25 or 29 mg PAN/P25-3 (to ensure the equivalent of P25) catalytic membrane was added in 50 mL of RhB (10 mg/L) solution, and the adsorption–desorption equilibrium state was reached after dark reaction for 30 min without turning on the light source. Then, we turned on the xenon lamp light source (A 300 W PLS-SXE300 xenon lamp, Beijing Perfect Light Technology Co., LTD, Beijing, China) for the degradation experiment. The suspension was taken every 30 min, and the absorbance A_t_ at 554 nm of the supernatant was recorded. The final residual quantity C_t_/C_0_ was approximated by A_t_/A_0_, where C_t_ and A_t_ represent the concentration and absorbance at time t, and C_0_ and A_0_ represent the initial concentration and absorbance. The degradation rate is calculated by (1 − C_t_/C_0_) × 100%.

### 2.5. Photocatalytic Disinfection of E. coli

Firstly, *E. coli* was inoculated in a Luria-Broth (LB) nutrient solution, cultured in a shaker at 37 °C for 12 h, centrifuged and collected, washed two times with sterile saline, and then re-suspended in fresh sterile saline. The final *E. coli* concentration was adjusted to 3 × 10^6^ CFU/mL. In a typical experiment, 10 mg of PAN/P25-3 nanotubular membrane was immersed into 10 mL of E. coil suspension. The photocatalytic sterilization performance of the PAN/P25-3 multi-porous nanotubular membrane was evaluated by a xenon lamp with a DT365 filter. Within a certain time interval, 500 μL suspension was taken and diluted with sterile saline. In order to determine the cell density of viable *E. coli*, 100 μL diluted solution was daubed to nutrient agar and incubated at 37 °C for 12 h. We then determined the number of viable bacteria. Each experiment was repeated three times.

### 2.6. Characterization

Fourier transform infrared (FT-IR) spectra were recorded on a Nicolet Instruments Research Series 5PC FTIR. The X-ray diffraction (XRD) patterns were obtained using a Shimadzu XRD-7000 X-ray diffractometer with monochromatized Cu Kα radiation. The XRD measurement was conducted with a 2θ range of from 10° to 80°, a scan speed of 5°/min, and a step size of 0.02°. The surface morphologies and composition of samples were examined using SEM (TESCAN MAIA 3 LMH, Bratislava, Czech Republic). TEM images were recorded by an FEI Tecnai F20 microscope operated at 200 kV. The Brunauer-Emmett-Teller (BET) specific surface area of the material was obtained by measuring the nitrogen adsorption–desorption isotherms using a nitrogen adsorption apparatus (ASAP2020, Norcross, GA, USA). Thermogravimetric (TG) was performed by heating the nanofiber membranes from 25 °C to 800 °C at a heating rate of 10 °C/min in the air atmosphere (Netzsch STA 449 F3, Selb, Bavaria, Germany). Static WCA tests for products were carried out via a contact angle meter (Krüss DSA25, Hamburg, Germany). The UV–vis diffused reflectance spectra (DRS) measurements of the samples were carried out using a TU-1901 system with BaSO_4_ as the reflectance standard. Photoluminescence (PL) spectra of the samples were detected on a fluorescence spectrophotometer (Hitachi F-7000, Tokyo, Japan) with an excitation wavelength of 200 nm. Electrochemical signals were recorded by a CHI660B electrochemical analyzer (Chenhua, Shanghai, China). Three-dimensional excitation–emission matrix (3DEEM) fluorescence spectra were recorded using a Fluoromax-4 spectrofluorometer (Zolix SmartFluo-Pro, Beijing, China), and both the excitation wavelength (Ex) and emission wavelength (Em) were in the range of 200–700 nm. The absorption spectra of organic dyes and antibiotics solutions were obtained by a UV–vis spectrophotometer (SHIMDZUUV-3600, Kyoto, Japan).

## 3. Results and Discussion

### 3.1. Composition and Crystal Structure

**Figure 2:**
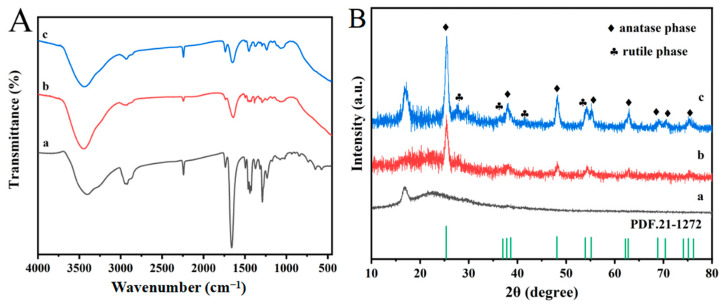
(**A**) FT-IR and (**B**) XRD patterns of (a) PAN/PVP; (b) PAN/PVP/P25-0.2; (c) PAN/P25-3.

In order to evaluate the physical and chemical changes of the composites, FT-IR and XRD are used for spectral analysis. Figure 2A shows the PAN/PVP fiber membrane, PAN/PVP/P25 fiber membrane and PAN/P25-3 multi-porous nanotubular membrane. Observing curve (a), the wide absorption peak near 3400 cm^−1^ is attributed to the stretching vibration of -OH, which is due to the adsorbed water on the sample surface. The absorption bands of the spectrum observed at 3000–2900 cm^−1^ and 1454 cm^−1^ are due to stretching and deformation vibrations of C-H bonds, respectively. At 2245 cm^−1^, the stretching vibration of the C≡N bond in PAN is presented [31,32,33,34]. The obvious peaks near 1450 cm^−1^ and 1250 cm^−1^ are indexed into C=C and C-N stretching vibrations. In addition, there is an obvious peak at 1680 cm^−1^, which is attributed to the C=O stretching vibration in the PVP molecule. Compared with curve (a), curves (b) and (c) have a new broad peak in 850–550 cm^−1^, which belongs to the Ti-O-Ti stretching bond and proves the existence of P25. Figure 2A (curve b) shows a decrease in the peak intensity at 1680 cm^−1^ for PAN/PVP/P25-0.2, even before the solvothermal process, which may suggest partial removal or changes in the PVP structure prior to the reaction. Figure 2B shows the XRD patterns. Curve (a) has no obvious peak. In curve (b), the anatase phase of P25 corresponds to 25.4°, 37.7°, 47.8°, 54.8°, 62.5° and 75° and the rutile phase of P25 corresponds to 27.3°, 36°, 41°, 53.8° and 74.9° [35,36], which confirms the existence of P25 in the composite film, but the peak intensity is weak. This is because most P25 nanoparticles are wrapped in the PAN fiber. Moreover, the XRD peak at approximately 2θ ≈ 17° observed in both the PAN/PVP and PAN/P25-3 patterns likely originates from the (100) reflection of the semi-crystalline structure of PAN. It is worth noting that the peak strength in curve (c) increases significantly. This is because more P25 nanoparticles are largely exposed to the surface of the PAN/P25 multi-porous nanotube after the removal of the PVP component, which is mainly ascribed to the role of the EG solvothermal induction process.

### 3.2. Morphology of PAN/P25 Multi-Porous Nanotubular Membrane

**Figure 3:**
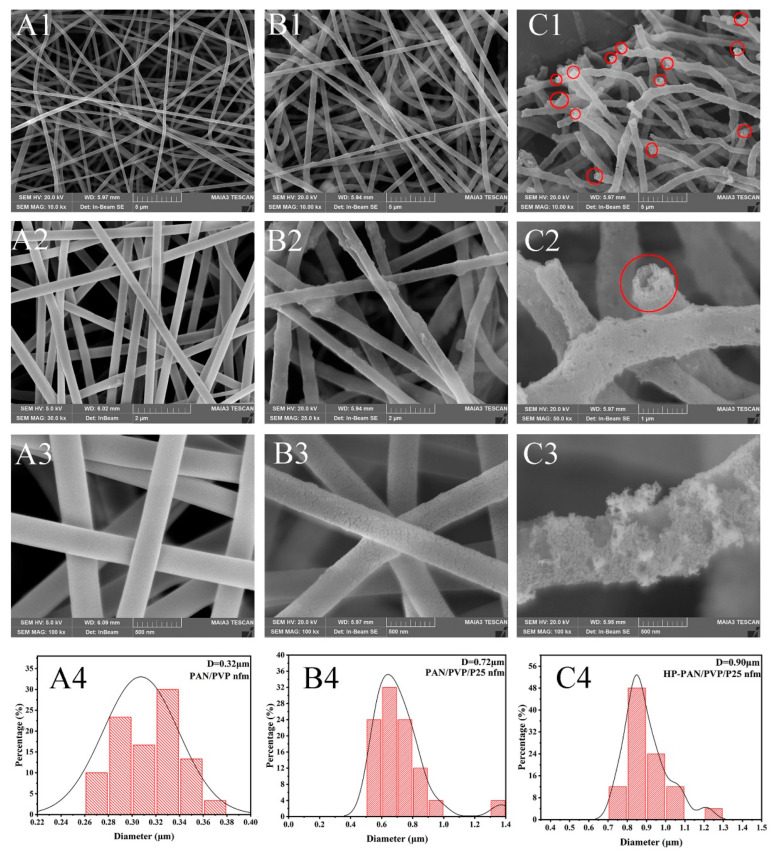
SEM images and diameter distribution of (**A1**–**A4**) PAN/PVP; (**B1**–**B4**) PAN/PVP/P25-0.2; (**C1**–**C4**) PAN/P25-3.

The surface morphology evolution of PAN/PVP fibrous membranes is observed by the SEM. As shown in Figure 3(A1–A4), the PAN/PVP fiber membrane possesses a smooth surface with a diameter of 0.32 μm. For the PAN/PVP/P25-0.2 fiber membrane in Figure 3(B1–B3), some P25 nanoparticles are uniformly loaded on the surface of the fiber membrane and the surface has become rough. Moreover, the fiber diameter increases from 0.32 μm to 0.72 μm because the viscosity of the spinning solution becomes larger, which makes it difficult for the electrostatic force to stretch the fiber more thoroughly. As depicted in Figure 3(C1–C4), it is interesting that the PAN/P25-3 multi-porous nanotubular structure is achieved after the ethylene glycol solvothermal induction treatment, the nanotubular structure is marked with a red circle, and there are many pores and P25 nanoparticles exposed to the surface of the PAN/P25 multi-porous nanotube (Red circle mark). The formation of multi-porous nanotubes is mainly dependent on the phase separation phenomenon between PAN and PVP [37]. Due to the different viscosity of the two components of PAN and PVP, the PAN component is mainly located outside the fibers, and the PVP component is mainly located inside the PAN/PVP fibers during the electrospinning process. Moreover, most of the PVP component is removed from the fibers due to the solubility of PVP in EG solvent during the EG solvothermal induction reaction. In addition, because PAN is rich in a large amount of -CN, which has the ability to trap metal ions, P25 is still preserved on the fiber. Moreover, the multi-porous nanotube diameter increases from 0.72 μm to 0.90 μm; the reason for this is that the removal of PVP leads to fiber swelling during the EG solvothermal treatment. The unique multi-porous nanotubular structure of PAN/P25-3 is favorable for the exposure of P25 nanoparticles and the light-harvesting and utilization by means of multiple reflections and scattering, yielding more electrons and holes. Furthermore, the multi-porous nanotubular structure can shorten the diffusion path of pollutants by passing through the hole in the tube wall and provide more active sites for contact with pollutants. As a consequence, the above two aspects are beneficial to the improvement of photocatalytic performance.

**Figure 4:**
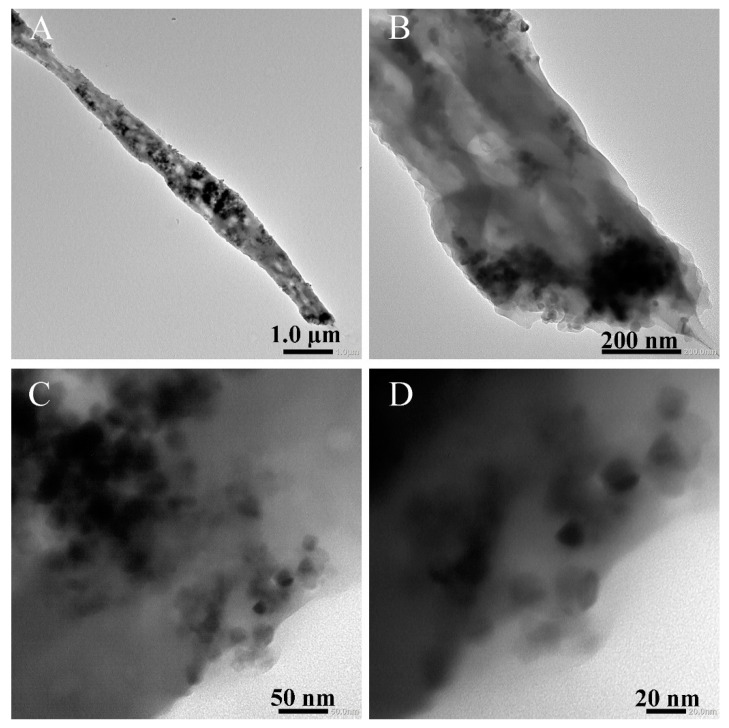
TEM images of PAN/P25-3 multi-porous nanotubular membrane.

TEM images in Figure 4A,B further confirm the one-dimensional and multi-porous hollow structure of PAN/P25-3. Moreover, it is vividly shown that the exposed P25 nanoparticles are located on the multi-porous nanotube surface in the enlarged TEM in Figure 4C,D. Both the increase in holes and the exposure of the P25 nanoparticles are beneficial to enhance the specific surface area and provide more active sites and flow channels, which can facilitate the organic dyes or bacteria in sewage contaction with the P25 photocatalyst. In addition, the light inside the holes can be refracted multiple times to improve the light utilization rate, thereby improving the photocatalytic efficiency.

### 3.3. Effect of Solvothermal Time on the Morphology of PAN/P25

**Figure 5:**
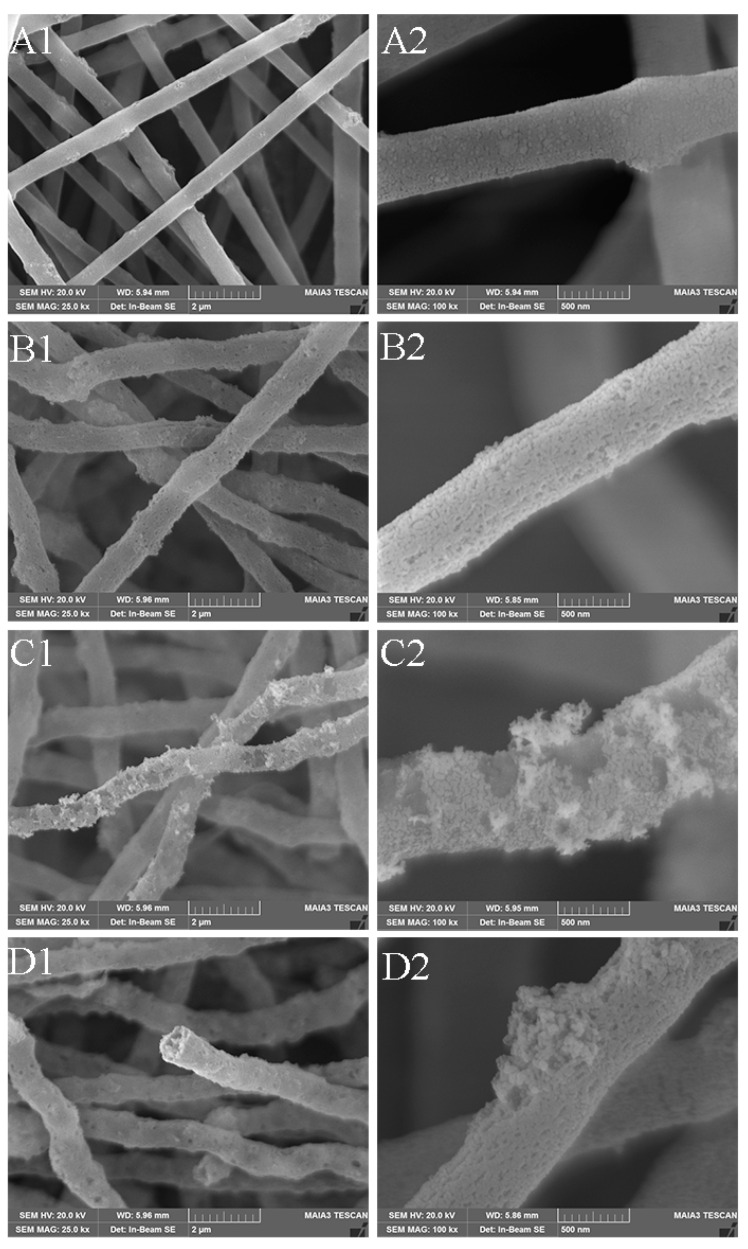
SEM images of (**A1**,**A2**) PAN/PVP/P25-0.2 and fiber membranes with different solvothermal reaction time (**B1**,**B2**) PAN/P25-2; (**C1**,**C2**) PAN/P25-3; (**D1**,**D2**) PAN/P25-4.

The surface morphology changes of the membranes at different times were observed by SEM. The PAN/PVP/P25-0.2 fibrous membrane without solvothermal treatment is shown in Figure 5(A1,A2); a small number of P25 nanoparticles are loaded on the surface of the fiber, most of them are coated inside the fiber, which makes it have a low photocatalytic efficiency. After solvothermal treatment for 2 h (Figure 5(B1,B2)), the holes on the surface of the fiber are attributed to the dissolution of PVP, but it does not cause the nanotubular structure, which is still unable to achieve the highest utilization of light. With the extension of the solvent heat treatment time, the pores on the surface of the fiber gradually increase, and more P25 is exposed while the fibers form a hollow tubular structure (Figure 5(C1,C2)). However, too long a time (4 h) leads to the agglomeration of nanoparticles P25 (Figure 5(D2)), which may affect the photocatalytic property.

### 3.4. Nitrogen Sorption Isotherms

**Figure 6:**
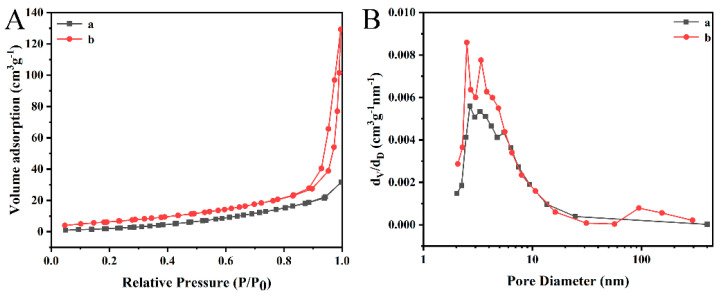
(**A**) N_2_ adsorption–desorption isotherms and (**B**) pore size distribution curves of (a) PAN/PVP/P25-0.2; (b) PAN/P25-3.

The physical properties of PAN/PVP/P25-0.2 and PAN/P25-3 are further characterized by the BET test. As shown in Figure 6, the N_2_ adsorption–desorption isotherms and pore diameter of PAN/PVP/P25-0.2 fiber membrane and PAN/P25-3 multi-porous nanotubular membrane were evaluated to determine their specific surface area and the pore size. The PAN/PVP/P25-0.2 fiber membrane exhibits a type III isotherm, which indicates that the surface of the material is a non-porous or macroporous material. The measured pores data are attributed to the interlacing pores between the fibers. The PAN/P25-3 multi-porous nanotubular membrane exhibits a type III isotherm and H_3_ type hysteresis loop (0.8 < P/P_0_ < 1.0), which is one of the main characteristics of mesoporous materials [38], and its pore size distribution is relatively narrow (2–20 nm). Table 2 shows the specific data; the surface areas of the PAN/PVP/P25-0.2 fiber membrane and the PAN/P25-3 multi-porous nanotubular membrane are 12.29 m^2^·g^−1^ and 25.74 m^2^·g^−1^, respectively. The average pore size increased distinctly, ranging from 15.33 nm to 24.76 nm, and the pore volume also increased significantly, from 0.047 cm^3^·g^−1^ to 0.160 cm^3^·g^−1^. This increase is attributed to the removal of PVP, which creates a large number of holes. These holes are beneficial for increasing the active sites and enhancing light absorption.

### 3.5. Thermal Analysis

**Figure 7:**
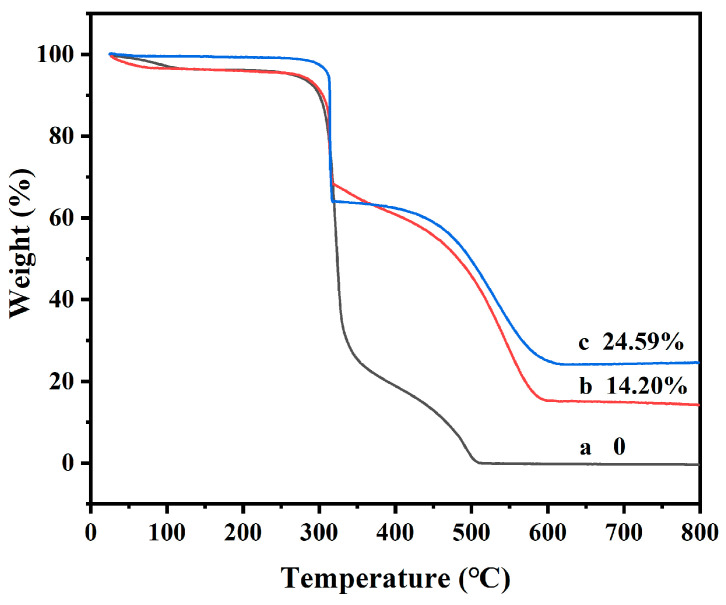
TG analysis of (a) PAN/PVP; (b) PAN/PVP/P25-0.2; (c) PAN/P25-3.

The TG analysis of the PAN/PVP fiber membrane, PAN/PVP/P25 fiber membrane and PAN/P25-3 multi-porous nanotubular membrane are shown in Figure 7. When the temperature reached 600 °C, PAN and PVP were all volatilized. As shown in observation curve (b), due to the hydrophilicity of PVP, the membrane surface adsorbs water in the air, and 3.41% of the mass loss around 100 °C is attributed to the water on the surface of the sample. The mass loss of 0.7% between 100 °C and 260 °C is attributed to residual DMF. The pyrolysis of PVP begins at 260 °C, and some PVP will cross-link with PAN [39]. At the same time, PAN is pyrolyzed at 280 °C. Therefore, 38.41% of the weight loss between 260 °C and 320 °C is ascribed to the partial decomposition of PVP and the loss of a small amount of ammonia and hydrogen cyanide during PAN cyclization [40]. Continuing to increase the temperature, the secondary weight loss of PAN starts due to carbonization and decomposition, but it is slower than the previous weight loss, which is due to the better heat resistance of PAN [41]. When the temperature rises to 600 °C, only P25 nanoparticles are left, and the residual weight of 14.20% (curve b) is close to the theoretical value (14.29%). Observing curve (c), due to the removal of PVP, the surface contains less adsorbed water, so there is almost no mass loss before 100 °C. The subsequent pyrolysis curve is similar to the trend of curve (a), and the final P25 content (24.59%) is close to the theoretical value (25%). It is suggested that the solvothermal process can remove the PVP component and hardly cause the shedding of P25 nanoparticles from the PAN/P25-3 multi-porous nanotubular membrane.

### 3.6. Wettability Test

**Figure 8:**
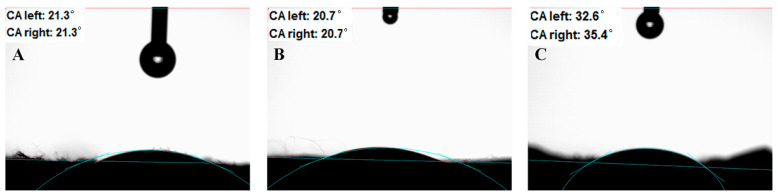
Water contact angle images: (**A**) PAN/PVP; (**B**) PAN/PVP/P25-0.2; (**C**) PAN/P25-3.

As shown in Figure 8, the wettability of the PAN/PVP fiber membrane, PAN/PVP/P25-0.2 fiber membrane and PAN/P25-3 multi-porous nanotubular membrane were evaluated by measuring the contact angle between the surface of the nanofiber membrane and water. Due to the hydrophilicity of PVP, the PAN/PVP fiber membrane shows good hydrophilicity, and the contact angle is only 21.3°. Although the surface of the fiber became rough after loading P25, it still shows good hydrophilicity in Figure 8B. The contact angle of the PAN/P25-3 multi-porous nanotubular membrane slightly increases to 35.4° and still shows hydrophilic properties; this is probably because some hydrophilic groups of -OH that originated from the EG solvent are introduced into the PAN/P25-3 surface during the solvothermal process. This hydrophilicity is beneficial to the treatment of pollutants in water. 

### 3.7. Optical Absorption and Photoelectrochemistry Analysis

**Figure 9:**
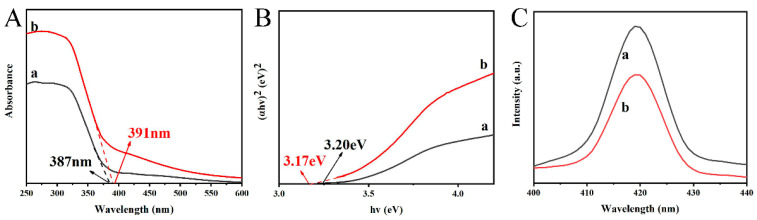
(**A**) DRS spectra; (**B**) plots of (αhν)^2^ versus energy (hν) and (**C**) PL spectra of (a) PAN/PVP/P25-0.2; (b) PAN/P25-3.

DRS was used to study the optical absorption behavior of the PAN/PVP/P25-0.2 fiber membrane and PAN/P25-3 multi-porous nanotubular membrane; the results are shown in Figure 9A. The absorption edges of the PAN/PVP/P25-0.2 fiber membrane and PAN/P25-3 multi-porous nanotubular membrane were at 387 nm and 391 nm, respectively, which is attributed to the intrinsic band gap absorption of P25 [42]. The higher light absorption value is because the porous structure is favorable for light to enter the inner wall, and the tubular structure is favorable for light to achieve multiple reflections in the inner wall, making the light utilization rate higher. At the same time, EG heat treatment may introduce functional groups such as -OH or -CHO to the surface of the material; this may also be another reason for the increased light absorption [20,22]. The obtained diffuse reflectance spectrum is converted into a Tauc diagram (Figure 9B) according to the following formula: αhν = A (hν − E_g_)^n/2^, Where α, h, v, A and E_g_ represent the absorption coefficient, Planck’s constant, optical frequency, direct leap constant and band gap energy, respectively. The value of n depended on the type of optical transition of semiconductors (n_direct_ = 1 and n_indirect_ = 4). The straight part of the figure is extended to the horizontal axis (y = 0) to obtain the band-gap energy (3.20 eV and 3.17 eV). The band-gap energy of the two samples did not change significantly, indicating that the P25 remained stable after solvent heat treatment.

It is well known that the recombination of photogenerated electrons (e^−^) and holes (h^+^) is one of the important parameters determining photocatalytic activity [43]. The PL spectra (Figure 9C) of the samples were detected at 200 nm excitation wavelength to compare and analyze the recombination rate of e^−^ and h^+^. It can be seen that the peak intensity of the PAN/P25-3 multi-porous nanotubular membrane at 420 nm was significantly lower than that of the PAN/PVP/P25-0.2 fiber membrane, which indicates that the photoinduced carrier separation efficiency was higher. This is because the recombination of e^−^ and h^+^ occurs not only within a single P25 molecule but also between two or more adjacent molecules. The porous structure may improve the dispersion of P25, thereby reducing the recombination of e^−^ and h^+^ between adjacent P25, thus reducing the recombination rate, and the multi-porous structure can shorten the diffusion path of e^−^ and h^+^, thus reducing the recombination rate.

### 3.8. Photoelectrochemical Properties Test

**Figure 10:**
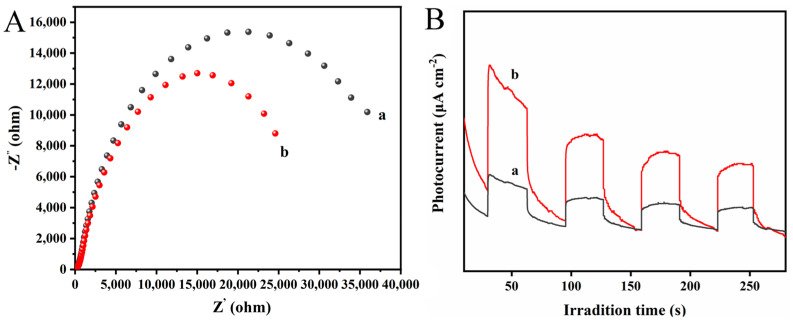
(**A**) EIS Nyquist plots and (**B**) photocurrents of (a) PAN/PVP/P25-0.2; (b) PAN/P25-3.

Figure 10 shows the photoelectrochemical properties of the membrane. The arc radius of the PAN/P25-3 multi-porous nanotubular membrane is smaller, indicating that it has less resistance to free charge migration and a faster charge transfer rate [44]. Faster charge transfer is beneficial to promote the separation efficiency of photogenerated carriers, thereby improving the photocatalytic efficiency. Moreover, compared with the PAN/PVP/P25-0.2 fiber membrane, the photocurrent intensity of the PAN/P25-3 multi-porous nanotubular membrane also increased, further confirming the effective separation of photo-generated e^−^ and h^+^ pairs.

### 3.9. Photodegradation Activity and Stability

**Figure 11:**
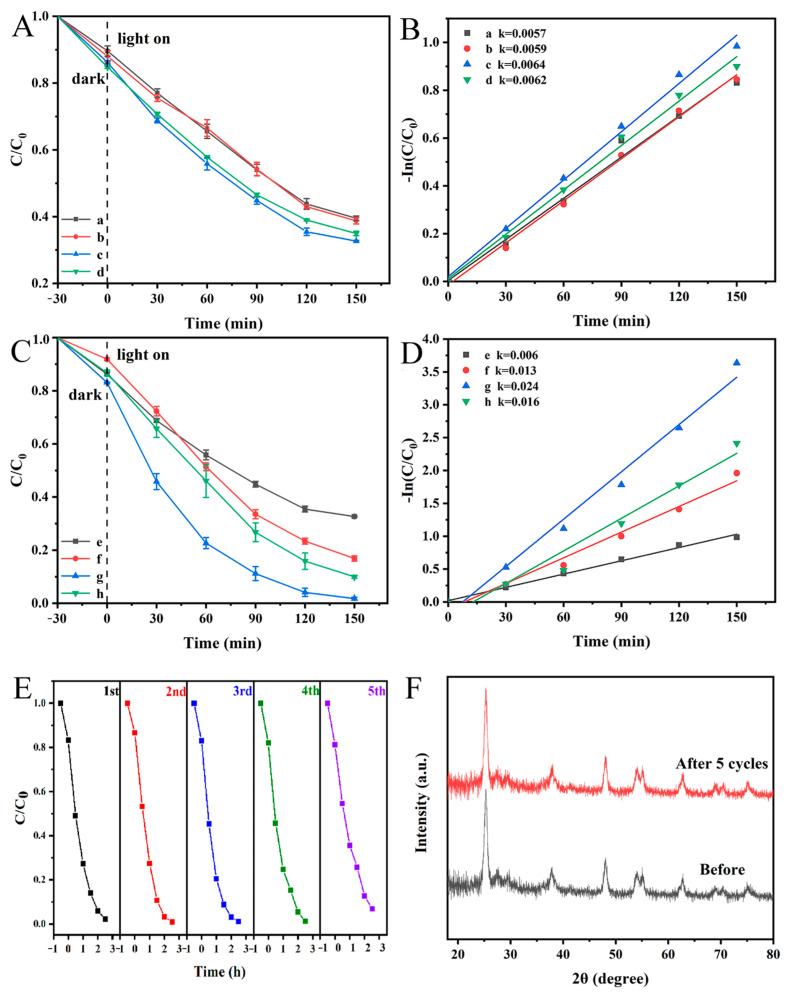
(**A**,**C**) Photocatalytic degradation and (**B**,**D**) kinetics curves for RhB aqueous solution over different photocatalysts under simulated solar light irradiation: (a) PAN/PVP/P25-0.1; (b) PAN/PVP/P25-0.15; (c,e) PAN/PVP/P25-0.2; (d) PAN/PVP/P25-0.25; (f) PAN/P25-2; (g) PAN/P25-3; (h) PAN/P25-4; (**E**) recycled photodegradation efficiency of PAN/P25-3; (**F**) XRD patterns of PAN/P25-3 before and after the recycling photocatalytic tests.

The performance of the obtained samples was studied by photocatalytic degradation of organic dyes, and RhB was selected as the object model. Figure 11 shows the photocatalytic degradation curve (A, C) and kinetic diagram (B, D) of RhB aqueous solution by different photocatalysts under simulated sunlight. All of the samples have degradation effects toward RhB, and the degradation characteristics conform to pseudo-first-order kinetics. The as-spun PAN/PVP/P25-0.2 fiber membrane was subjected to EG solvothermal treatment for different durations (2 h, 3 h and 4 h), resulting in the formation of multi-porous nanotubular membranes: PAN/P25-2, PAN/P25-3, and PAN/P25-4. These membranes exhibited improved photodegradation performance compared to the PAN/PVP/P25-0.2 membrane (Figure 11C), as the PAN/P25 multi-porous nanotubular membranes have more exposed P25 particles. In contrast, most of the P25 nanoparticles are incorporated within the fibers of the PAN/PVP/P25-0.2 membrane. With increasing treatment time, the photocatalytic degradation efficiency followed the following order: PAN/P25-3 > PAN/P25-4 > PAN/P25-2. Among them, the PAN/P25-3 multi-porous nanotubular membrane demonstrated the highest photocatalytic degradation efficiency of 96.1% for RhB within 120 min. According to the literature [45], the greater the rate constant k, the stronger the photocatalytic performance of the photocatalyst. The degradation rate (0.0240 min^−1^) of the PAN/P25-3 multi-porous nanotubular membrane is four times that of the PAN/PVP/P25-0.2 fiber membrane (0.006 min^−1^). This is because the multi-porous nanotubular membrane PAN/P25-3 has a larger specific surface area, higher light absorption, lower e^−^-h^+^ recombination rate, smaller impedance and stronger photocurrent.

Stability is an important index affecting the practical application of the material [11]. PAN/P25-3 is subjected to five photocatalytic cycles. The used catalyst was removed, and the residual dye on the surface was washed with anhydrous ethanol and deionized water and then reused after freeze-drying. Figure 11E depicts the recycled photocatalytic performance of the PAN/P25-3 photocatalyst, which keeps its original properties of about 94% even after five runs. In addition, the XRD results (Figure 11F) show that the basic composition and structure have almost no change before and after use, and there was no significant change in mass before and after the reaction, which demonstrates that the PAN/P25-3 multi-porous nanotubular membrane has excellent stability.

### 3.10. Photocatalytic Process and Mechanism

**Figure 12:**
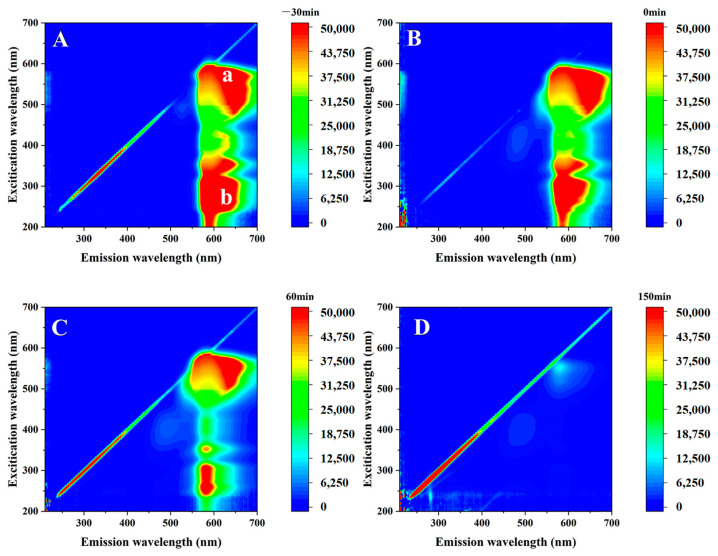
The 3D-EEM fluorescence spectra of RhB degradation by PAN/P25-3: (**A**) −30 min; (**B**) 0 min; (**C**) 60 min; (**D**) 150 min.

The photocatalytic PAN/P25-3 degradation process of the RhB solution was studied by the 3D-EEM fluorescence method. Initially, molecular fluorescence is attributed to the rigid planar structure of RhB, which can accelerate the conjugation effect of π electrons and improve fluorescence efficiency [46,47]. As shown in Figure 12A, the two fluorescence characteristic peaks of RhB after the adsorption–desorption equilibria are the (a) peak at Ex/Em of 550–575/620–660 nm and (b) peak at 225–275/575–650 nm [48]. The 3D-EEM fluorescence spectrum after adsorption equilibrium is shown in Figure 12B. The shape, position and intensity of the fluorescence characteristic peak are almost unchanged, indicating that RhB molecules are not decomposed during this process, just adsorbed on the surface of the photocatalyst. With the photocatalytic degradation time of 60 min (Figure 12C), the position of the fluorescence characteristic peak exhibits gradually red-shifted, indicating that the RhB molecules are decomposed. This phenomenon may be related to the generation of N-deethylated intermediates, in which macromolecules are converted into relatively small fragments and specific functional groups such as amines, hydroxyl groups and carbonyl groups are removed [49]. In addition, when the visible light irradiation time was extended to 150 min (Figure 12D), the intensity of the fluorescence characteristic peak in the residual solution gradually decreased, indicating that the formed n-deethylation intermediate may be further decomposed into small molecules. Therefore, the main process of RhB degradation is the dissociation of the conjugated chromophore structure of the RhB molecule and the mineralization of the corresponding intermediates.

**Figure 13:**
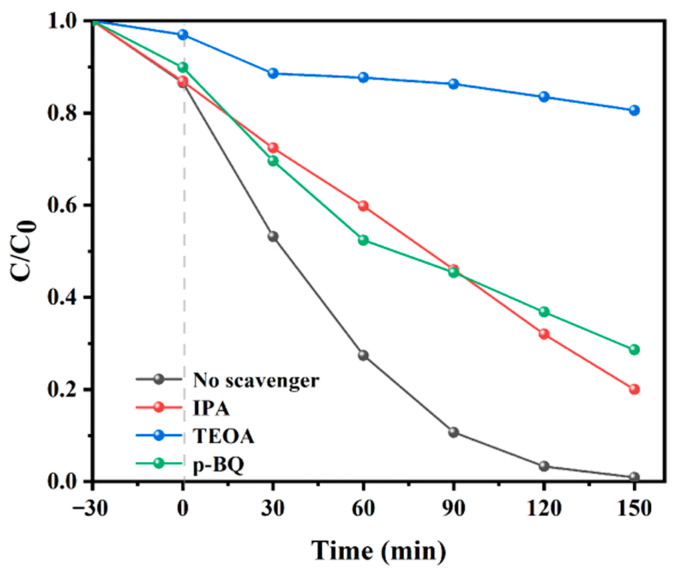
The active species trapping experiments during the photocatalytic degradation of RhB over PAN/P25-3 multi-porous nanotubular membrane.

In order to further explain the photocatalytic mechanism of the PAN/P25-3 multi-porous nanotubular membrane, triethanolamine (TEOA), p-benzoquinone (p-BQ) and isopropanol (IPA) were used as the capture agents of photogenerated holes (h^+^), superoxide radicals (·O_2_^−^) and hydroxyl radicals (·OH), respectively. As shown in Figure 13, the catalytic efficiency of PAN/P25-3 decreased to different degrees after the addition of three free radical trapping agents. These results show that the three kinds of free radicals have different effects on photocatalytic efficiency. Among them, the degradation rate of RhB is significantly reduced after TEOA is added, indicating that h^+^ has been captured successfully, leading to a decrease in photocatalytic activity. However, the degradation rate of RhB decreases slightly in the presence of IPA and p-BQ, indicating that ·OH and ·O_2_^−^ are not the main active species. In this case, it can be determined that h^+^ is the main active species for the degradation of RhB by PAN/P25-3 multi-porous nanotubular membrane. ·O_2_^−^ and ·OH are the secondary active substances.

### 3.11. Bacterial Inactivation Activity

The PAN/PVP fibrous membrane was initially obtained using the direct electrospinning method. When 0.2 g of P25 particles was dispersed in the PAN/PVP spinning solution, the PAN/PVP/P25-0.2 fibrous membrane was produced. The as-spun PAN/PVP/P25-0.2 fibrous membrane was then subjected to the EG solvothermal induction process for 3 h, resulting in the flexible PAN/P25-3 multi-porous nanotubular membrane. As shown in Figure 11C, PAN/P25-3 exhibited better photocatalytic degradation performance than the PAN/PVP/P25-0.2 membrane, which led to the selection of PAN/P25-3 for further investigation of its bacterial inactivation activity.

**Figure 14:**
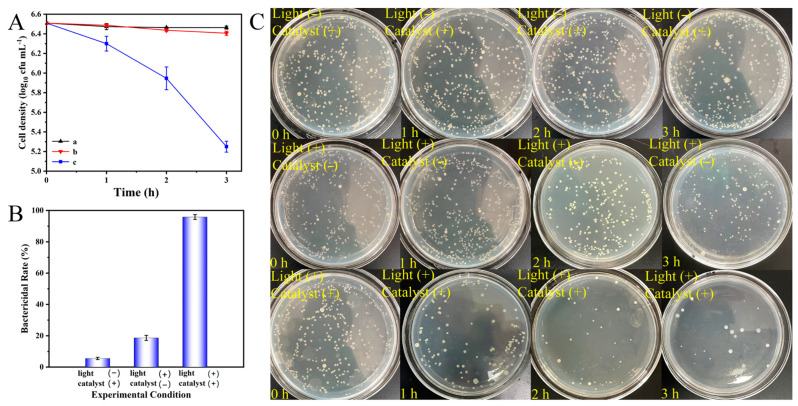
Photocatalytic bactericidal activity of PAN/P25-3 multi-porous nanotubular membrane with a dilution factor of 5000: (**A**) sterilization line under different conditions: (a) only PAN/P25-3; (b) only 365 nm light; (c) PAN/P25-3 with 365 nm light; (**B**) bactericidal rate of *E. coli* and (**C**) optical image of *E. coli* colonies.

In addition, we also examined the photocatalytic sterilization performance of the PAN/P25-3 multi-porous nanotubular membrane under 365 nm light irradiation, as shown in Figure 14. Each experiment was repeated three times to ensure reliability and reproducibility. Under dark conditions, the survival number of *E. coli* cells does not decrease significantly after 3 h, which indicates that the material itself is non-toxic to *E. coli* and has just a weak adsorption. When only light irradiation at a wavelength of 365 nm is applied, the bacterial concentration decreases by 0.1 log within 3 h; this is because the band of light can make the bacterial oxygen free radicals, which cause oxidative stress and lead to bacterial death [50]. However, when the PAN/P25-3 multi-porous nanotubular membrane is used under light conditions, the inactivation rate of *E. coli* is significantly improved, and the bactericidal rate of *E. coli* is over 95%, which is obviously higher than the sum of the inactivation rate under light irradiation alone and only catalyst. It is indicated that the synergistic effect of the PAN/P25-3 multi-porous nanotubular membrane with light irradiation may play an important role. Under light conditions, PAN/P25-3 can produce different active free radicals, such as h^+^, e^−^, ·O_2_^−^, etc. Where h^+^ and e^−^ can initiate redox reactions, and both can be converted into hydroxyl radicals, hydroxyl radicals can quickly attack and seize hydrogen atoms on viral and bacterial proteins or envelopes, inducing them to lose their normal physiological functions and leading to death. ·O_2_^−^ attacks on bacteria can cause a decrease in the activity of some antioxidant enzymes (catalase, superoxide dismutase) of bacteria and damage cell membranes, resulting in gradual oxidative damage and leakage of intracellular substances, especially proteins and DNA, the similar mechanism of photocatalytic bacterial inactivation is similar to that in reference [51].

## 4. Conclusions

In summary, a flexible PAN/P25 multi-porous nanotubular membrane was successfully prepared by the electrospinning and solvothermal methods. We explored the optimal spinning parameters and solvothermal reaction time. Interestingly, the solvothermal reaction not only transforms the fiber into a multi-porous nanotubular structure but also exposes the P25 wrapped in the fiber to the outside of the fiber. Compared with the PAN/PVP/P25-0.2 fiber membrane, the photocatalytic RhB degradation activity of the PAN/P25-3 multi-porous nanotubular membrane increased by four times. This enhanced photocatalytic performance can be attributed to the expanded specific surface area, more active edge exposure, higher light absorption efficiency, high light utilization, smaller impedance and stronger photocurrent response. After five cycles, the material still maintains good stability, which will be well applied in the environmental field. At the same time, the material also has photocatalytic sterilization ability; under 365 nm monochromatic light irradiation for 3 h the inactivation rate of *E. coli* was as high as 95.8%.

## Figures and Tables

**Table 1:** Recipes of PAN/P25 multi-porous nanotubular membranes.

Sample	PAN (g)	PVP (g)	P25 (g)	Solvothermal Time (h)
PAN/PVP/P25-0.1	0.6	0.6	0.1	—
PAN/PVP/P25-0.15	0.6	0.6	0.15	—
PAN/PVP/P25-0.2	0.6	0.6	0.2	—
PAN/PVP/P25-0.25	0.6	0.6	0.25	—
PAN/PVP	0.6	0.6	0	—
PAN/P25-2	0.6	—	0.2	2
PAN/P25-3	0.6	—	0.2	3
PAN/P25-4	0.6	—	0.2	4

**Table 2:** Physical properties of the photocatalytic membrane.

Sample	S_BET_ (m^2^·g^−1^)	Average Pore Size (nm)	Pore Volume (cm^3^·g^−1^)
PAN/PVP/P25-0.2	12.29 ± 0.01	15.33 ± 0.01	0.047 ± 0.001
PAN/P25-3	25.74 ± 0.01	24.76 ± 0.01	0.160 ± 0.001

## Data Availability

The data presented in this study are available on request from the corresponding author.
